# Antibacterial efficacy of nano-silver, nano-chitosan and silver diamine fluoride against *Streptococcus mutans*

**DOI:** 10.6026/973206300223043

**Published:** 2026-05-31

**Authors:** Imran Md, Shivam Sulok, Karuna Bharti, Kalpana Thakur, Rudra Mazumdar, Abhishek Sinha

**Affiliations:** 1 Department of Dentistry, Patna Medical College & Hospital, Patna, Bihar, India; 2 Department of Prosthodontics and crown & bridge, BJSDCH, Ludhiana, Punjab, India; 3 Department of Conservative Dentistry & Endodontics, MMDCH, Darbhanga, India; 4 Department of Paediatric Dentistry & Preventive Dentistry, MMDCH, Darbhanga, India; 5 Department Conservative Dentistry & Endodontics, HIDSAR, Haldia, India

**Keywords:** Nano silver, nano-chitosan, silver diamine fluoride, *Streptococcus mutans*, primary teeth, antibacterial

## Abstract

Dental caries in primary teeth remains a major pediatric health concern driven by *Streptococcus mutans*, necessitating effective antimicrobial strategies. Therefore, it is of interest to compare the antibacterial efficacy of nano silver, nano-chitosan and silver diamine fluoride against *S. mutans* isolated from carious primary teeth. Hence, an *in vitro* design using agar well diffusion, minimum inhibitory concentration and minimum bactericidal concentration assays was conducted on samples from 30 extracted primary molars. Nano silver demonstrated the highest antibacterial activity followed by silver diamine fluoride and nano-chitosan, with the lowest MIC observed for nano silver. Thus, we show that nano silver exhibits superior antibacterial potential and may serve as an effective adjunct in pediatric caries management.

## Background:

Early childhood caries is still one of the most common chronic infectious diseases of the pediatric age group worldwide, as the prevalence rate of the condition is reported to vary between 23 and 90 percent in children across countries and socioeconomic stratums and children in developing countries and underserved populations have disproportionate rates of the disease [1]. The disease is typified by the rapid loss of primary tooth structure; the initial loss is an enamel demineralization followed by the dentin loss up to the ultimate pulpal compromise resulting into severe pain, sepsis, nutritional deficiency, impaired speech development and poor psychological impacts on affected children [2]. In spite of the improved preventative dentistry, early childhood caries has remained a daunting challenge in its management, due to the fact that the target population, which is the children below the age of six years, often has a low ability to cooperate with conventional restorative procedures, thus necessitating the development of minimally invasive and chemotherapeutic modalities of treating caries [3]. *Streptococcus mutans* has been continually cited as the key etiological factor in the initiation and progression of caries in the mouth by its distinctive constellation of virulence factors such as acidogenicity, aciduricity, the use of glucosyltransferase to generate glucans to support biofilm formation and the ability to store intracellular polysaccharides that facilitate the maintenance of acid production even in the absence of substrates [4]. It colonizes the surface of the tooth in the dental biofilm and the metabolic activity of the organism develops a localized acidic microenvironment that promotes demineralization of enamel and dentin hydroxyapatite, resulting in formation of a clinically visible cavitated lesion [5]. Successful elimination of the *S. mutans* populations on the tooth surface and inside caries lesions is, consequently, one of the main therapeutic goals in caries prevention and arrest interventions. Silver diamine fluoride has become a paradigm-shifting agent in the management of childhood caries, with regulatory acceptance and a high degree of clinical application as an effective noninvasive topical agent in the prevention of carious activity in primary teeth [6]. The two-pronged effect of the antibacterial nature of the silver ions as well as the remineralizing nature of fluoride, offers antimicrobial and mineral restorative effect. Clinical trials show arrest rates of 66% to 91% in treated carious lesions in primary teeth in which silver diamine fluoride has been used as an evidence-based alternative to standard restorative care in groups with limited access to dental care or in which behavioral control of young children proves to be a difficult task [7]. Nevertheless, silver diamine fluoride is linked with the clinically relevant drawback of permanent staining of treated carious tissue with the colors black, unlike roots of esthetic concerns among parents and this might limit acceptability, especially in the anterior teeth [8].

The advent of nanotechnology-based antimicrobial agents has created new opportunities in the creation of antibacterial materials of the dental applications. Nano silver particles, with a diameter size of between 1 and 100 nanometers, have significantly better antibacterial effects than the bulk counterparts because of the highly increased surface area-to-volume ratio, which increases the release of bioactive silver ions as well as to direct interactions between nanoparticles and cell membranes [9]. Nano silver has multifaceted antibacterial mechanisms involving disruption of bacterial cell membrane integrity by electrostatically attracting negatively charged phospholipid components, formation of reactive oxygen species leading to damage of proteins and DNA of intracellular organelles and interference with the functioning of the electron transport chain as well as inhibition of critical enzymatic processes [10]. Improved results through these various concurrent action mechanisms have resulted in the bacterial resistance development being significantly less possible than the traditional single-target antibiotics [11]. A naturally derived biopolymer, chitosan, is a compressed form of chitin found in crustacean exoskeletons and is currently getting a lot of interest as an antimicrobial biomaterial, because it has inherent antibacterial properties, excellent biocompatibility, biodegradability and mucoadhesive attributes [12]. The antimicrobial effect of chitosan can be mainly connected to the electrostatic interaction of the cationic amino group on the chitosan molecules and the negatively charged parts of bacterial cell membranes and cell walls leading to the increased permeability of the membranes, leakage of intracellular molecules and cell death [13]. Nano-chitosan also exhibits better antibacterial activity than the traditional chitosan when nano-sized particles are obtained because they possess a higher density of the surface charge and a higher ability to interact with the bacterial cell surface given the larger surface area to volume ratio [14]. Moreover, nano-chitosan has some benefits such as natural source, no oral discoloration, known biocompatibility with tissues and its capacity to act as a carrier of other therapeutic agents [15]. Although each of these three agents has been evaluated individually regarding its antibacterial activity against oral pathogens, there is little direct comparative analysis of any of the agents under standardized conditions. The majority of currently available studies have compared these agents to laboratory reference strains of *S. mutans*, which might not be reflective of the phenotypic traits and antimicrobial susceptibility phenotypes of real clinical isolates of carious primary teeth [16]. Active caries isolates can be more virulently active and have an ability to form biofilms and be resistant to antimicrobials than reference isolates, so their utilization in antibacterial assays will be more clinically relevant [17]. Therefore, it is of interest to compare the antibacterial efficacy of nano silver, nano-chitosan and silver diamine fluoride against *Streptococcus mutans* in primary teeth under standardized *in vitro* conditions.

## Materials and Methods:

## Study design:

This comparative *in vitro* microbiological study was conducted at the Department of Pedodontics and Preventive Dentistry in collaboration with the Department of Microbiology, over a period of six months from July 2025 to December 2025.

## Sample collection and bacterial isolation:

Thirty carious primary molars extracted for therapeutic reasons from children aged 4 to 9 years attending the pediatric dental clinic were collected following informed parental consent. Teeth were included if they exhibited clinically evident active dentin caries without pulpal involvement and excluded if they had previously received any restorative treatment, topical fluoride application within the preceding month, or if the child had received systemic antibiotic therapy within the preceding four weeks.

Immediately following extraction, each tooth was placed in a sterile container with Brain Heart Infusion broth (HiMedia Laboratories, Mumbai, India) and transported to the microbiology laboratory within one hour. Carious dentin samples were aseptically excavated using sterile spoon excavators under laminar airflow conditions. The excavated dentin was placed in 1 mL of sterile phosphate-buffered saline and vortexed for 60 seconds to create a homogeneous suspension. Serial ten-fold dilutions of the suspension were prepared and 100 μL of each dilution was inoculated onto Mitis Salivarius Bacitracin agar plates (selective medium for *S. mutans*), which were incubated anaerobically at 37°C for 48 hours in an anaerobic jar using gas-generating sachets (AnaeroGas Pack, HiMedia). Characteristic *S. mutans* colonies - raised convex, undulated, adherent colonies with a frosted glass appearance - were identified and subcultured onto fresh Brain Heart Infusion agar plates for purification.

## Bacterial identification and confirmation:

Isolated colonies were subjected to confirmatory identification through:

[1] Gram staining (gram-positive cocci in chains)

[2] Catalase test (catalase-negative)

[3] Biochemical tests including mannitol fermentation, sorbitol fermentation, arginine hydrolysis (negative) and esculin hydrolysis

[4] Growth characteristics on Mitis Salivarius Bacitracin agar

Following confirmation, a single representative *S. mutans* isolate was selected and maintained on BHI agar slants at 4°C for use in all subsequent antibacterial assays. A reference strain of *S. mutans* (ATCC 25175) was also obtained and tested in parallel as a quality control organism.

## Preparation of antimicrobial agents:

[1] Nano silver particles (AgNPs): Commercially synthesized silver nanoparticles (Sigma-Aldrich, St. Louis, MO, USA; catalog #730785) with a mean particle size of 20 nm (range 15-30 nm), spherical morphology and purity ≥ 99.5% were used. Nanoparticle characterization was verified through dynamic light scattering (Malvern Zetasizer Nano ZS) confirming mean hydrodynamic diameter of 24.6 ± 3.8 nm and zeta potential of -28.4 ± 2.1 mV. Stock suspension was prepared at 1000 μg/mL in deionized water and sonicated for 30 minutes before each use.

[2] Nano-chitosan particles (NCS): Chitosan nanoparticles were synthesized through the ionic gelation method using low molecular weight chitosan (deacetylation degree ≥ 85%, Sigma-Aldrich) and sodium tripolyphosphate as the cross-linking agent. Chitosan (0.2% w/v) was dissolved in 1% acetic acid and sodium tripolyphosphate solution (0.1% w/v) was added drop wise under magnetic stirring at room temperature. The resulting nanoparticle suspension was centrifuged at 12,000 rpm for 30 minutes and the pellet was resuspended in deionized water. Particle characterization by dynamic light scattering revealed a mean particle size of 148.3 ± 22.6 nm and zeta potential of 32.7 ± 3.4 mV. Stock suspension was prepared at 1000 μg/mL.

[3] Silver diamine fluoride (SDF): A commercially available 38% silver diamine fluoride solution (Advantage Arrest, Elevate Oral Care, West Palm Beach, FL, USA) was used. For experimental assays, the stock solution (approximately 380,000 μg/mL Ag) was serially diluted in sterile deionized water to achieve working concentrations equivalent to those used for the other test agents.

Three working concentrations were prepared for each agent: 500 μg/mL (high), 250 μg/mL (medium) and 125 μg/mL (low). All solutions were freshly prepared on the day of testing under aseptic conditions.

## Agar well diffusion assay:

Standardized bacterial suspensions were prepared by adjusting overnight BHI broth cultures of the clinical *S. mutans* isolate to 0.5 McFarland standard (approximately 1.5 x 10,^8^ CFU/mL) using a densitometer. Mueller-Hinton agar plates supplemented with 5% defibrinated sheep blood were prepared and inoculated by swabbing the standardized bacterial suspension uniformly across the entire plate surface in three directions. Wells of 6 mm diameter were aseptically punched into the agar using a sterile cork borer. Fifty microliters of each antimicrobial agent at each concentration were dispensed into designated wells. A well containing sterile deionized water served as the negative control. Plates were prepared in triplicate for each concentration of each agent. After 30 minutes of pre-diffusion at room temperature, plates were incubated anaerobically at 37°C for 24 hours. Zones of inhibition (ZOI) were measured in millimetres using a digital caliper with 0.01 mm accuracy, recording the mean of two perpendicular diameter measurements for each zone. The entire experiment was performed three times independently.

## Minimum inhibitory concentration (MIC) determination:

MIC was determined using the broth microdilution method in accordance with Clinical and Laboratory Standards Institute guidelines. Two-fold serial dilutions of each antimicrobial agent were prepared in BHI broth in sterile 96-well flat-bottom microtiter plates, yielding final concentrations ranging from 1000 μg/mL to 3.9 μg/mL. Each well received 100 μL of diluted antimicrobial agent and 100 μL of standardized bacterial suspension (adjusted to achieve a final inoculum of approximately 5 x 10^5^ CFU/mL). Positive control wells (broth + bacteria, no antimicrobial) and negative control wells (broth + antimicrobial, no bacteria) were included. Plates were incubated anaerobically at 37°C for 24 hours. MIC was defined as the lowest concentration at which no visible turbidity was observed. Turbidity was confirmed by spectrophotometric measurement at 600 nm using a microplate reader. All tests were performed in triplicate.

## Minimum bactericidal concentration (MBC) determination:

To determine MBC, 10 μL aliquots from wells at and above the MIC concentration showing no visible growth were subcultured onto fresh BHI agar plates and incubated anaerobically at 37°C for 48 hours. MBC was defined as the lowest concentration at which no colony growth was observed on the subculture plates, indicating ≥ 99.9% killing of the original inoculum. Tests were performed in triplicate.

## Time-kill kinetics assay:

Time-kill kinetics was assessed at 2x MIC concentration for each agent. Bacterial suspensions (5 x 10^5^ CFU/mL) were exposed to each antimicrobial agent and viable counts were determined at 0, 1, 2, 4, 6, 12 and 24 hours by serial dilution and plating on BHI agar. Colony-forming units per millilitre were calculated at each time point. Tests were performed in duplicate.

## Statistical analysis:

Data were analyzed using SPSS version 26.0 (IBM Corp., Armonk, NY, USA). Descriptive statistics were expressed as mean ± standard deviation. One-way ANOVA was used to compare ZOI values among agents at each concentration. Two-way ANOVA was used to assess the effects of agent type and concentration on ZOI. Post-hoc Tukey's HSD test was used for pairwise comparisons. The Kruskal-Wallis test was used for MIC and MBC comparisons. Time-kill data were analyzed using repeated measures ANOVA. Statistical significance was set at p < 0.05.

## Results:

*Streptococcus mutans* was successfully isolated from 27 of the 30 carious primary molars (isolation rate: 90.0%). All isolates demonstrated consistent morphological, cultural and biochemical characteristics confirming identification as *S. mutans*. One representative clinical isolate demonstrating robust growth characteristics was selected for all subsequent assays. Results obtained with the ATCC reference strain showed similar trends to those obtained with the clinical isolate, validating the experimental methodology. The zones of inhibition for all three antimicrobial agents at three concentrations are presented in Table 1. All agents demonstrated dose-dependent antibacterial activity with significantly increasing ZOI values at higher concentrations. Nano silver produced the largest zones of inhibition at all three concentrations, followed by silver diamine fluoride and nano-chitosan. Two-way ANOVA confirmed significant main effects for both agent type (F = 124.67, p < 0.001) and concentration (F = 198.34, p < 0.001), with a non-significant interaction (F = 1.82, p = 0.134), indicating that the rank order of antibacterial efficacy was consistent across concentrations. Post-hoc Tukey's HSD test revealed significant differences between all agent pairs at every concentration level (all p < 0.01). The minimum inhibitory concentration and minimum bactericidal concentration values for all three agents against both the clinical isolate and the reference strain are presented in Table 12. The MIC values followed the same rank order as the agar diffusion results, with nano silver demonstrating the lowest MIC (31.25 μg/mL), followed by SDF (62.5 μg/mL) and nano-chitosan (125 μg/mL). The MBC/MIC ratio of 2.0 for all three agents indicated bactericidal rather than merely bacteriostatic activity. Clinical isolates demonstrated consistently higher MIC and MBC values compared to the ATCC reference strain (approximately two-fold), suggesting reduced susceptibility in the clinical strain. The time-kill kinetic profiles at 2x MIC concentration are presented in Table 3. Nano silver achieved complete bactericidal activity (no detectable viable organisms) by 12 hours, SDF by 24 hours and nano-chitosan showed near-complete killing by 24 hours with residual low-level viability. Repeated measures ANOVA confirmed significant differences in the rate of bacterial killing among the three agents (F = 86.42, p < 0.001), with post-hoc analysis demonstrating significant pairwise differences at all-time points from 2 hours onward (all p < 0.05).

## Discussion:

The current research is the first recorded head-to-head study involving nano silver, nano-chitosan, silver diamine fluoride against *Streptococcus mutans* that are isolated in carious primary teeth by using various complementary antibacterial evaluation systems. The findings indicate that all the three agents have a high antibacterial action on *S. mutans* with nano silver having the best potency compared to silver diamine fluoride and nano-chitosan, hence violating the null hypothesis. The high rate of antibacterial performance that superiority of nano silver particles showed in all the three evaluation methods can be attributed to the special physicochemical characteristics that their nanosize endow. With the mean particle diameter of about 20 nm, the silver nanoparticles in the present study have an enormous surface-to-volume ratio, which maximises the amount of silver atoms at the particle surface and ensures a rapid dissolution and release of bactericidal Ag + ions into the medium surrounding the particles [18]. Small particle size also allows direct penetration of the bacterial cell wall and membrane, which is another form of contact-mediated killing mechanism that is not dependent on ion release. It's very negative zeta potential (-28.4 mV) is the primary factor of colloidal stability and inhibits aggregation of the particles and preserves the maximum possible surface area of the particles to interact with the antibacterial during the exposure period [19]. The various synergistic ways in which nano silver may act as antibacterial agent which include membrane disruption, intracellular reactive oxygen species generation, ribosomal destabilization and interference with DNA replication are what combine to present a multi-faceted antimicrobial attack that serves as attribution to the lower MIC values and faster killing kinetics when compared to the other agents [20]. The absolute sterilization of time-kill of 12 hours is further evidence of the bactericidal activity of nano silver at concentrations merely two-fold that of the MIC, which is clinically viable at the topical level of dentistry since long-term therapeutic levels can be achieved at the tooth surface. Silver diamine fluoride had intermediate antibacterial activity and the MIC values were precisely twice those of nano silver. The SDF antibacterial activity is through release of silver ions of the diamine silver fluoride complex that reacts with cell constituents of bacteria in the same way as the ionic silver of nanoparticles released [21]. The silver in SDF is however in an ionic form with ammonia ligands as opposed to the metallic nanoparticles and the kinetics and bioavailability of silver ions may vary between nanoparticulate silver and silver ions. Although the fluoride in the SDF may not have an antimicrobial effect in itself but instead reduces bacterial enolase and disrupts the glycolytic metabolism, the fluoride element might act as an additional antimicrobial effect [22]. The clinical benefit of SDF is that it has dual action wherein it offers antibacterial and remineralizing actions at the same time as opposed to nano silver by itself. Nano-chitosan showed the least antibacterial activity of the three agents yet showed a clinically significant activity with a value of MIC of 125 μg/mL. The antimicrobial action of the chitosan nanoparticles is based on its naturally different antimicrobial mechanism with the silver based agents, which is based on the establishment of the electrostaticly disturbed negatively charged bacterial cell membrane by the positively charged amino groups on the chitosan polymer backbone [23]. The availability of the surface charge in the electrostatic interaction is confirmed by the positive zeta potential of the nano-chitosan particles (+32.7 mV). Nevertheless, the contact-dependent agent of chitosan antibacterial action can make its application less effective than the antibacterial agents such as nano silver which can act both via contact-dependent and ion-release-dependent mechanisms [24]. The results of the observation using clinical isolates showing about two-fold greater MIC and MBC values in comparison to the ATCC reference strain in all three agents are relevant clinical findings. Active caries clinical isolates can express increased virulence factors, altered cell surface properties as well as possibly developed tolerance defenses which makes them less vulnerable to antimicrobial effectors than the laboratory-adapted reference strains [25]. This observation highlights the usefulness of clinical isolates of the target patient population in the antimicrobial efficacy study to provide clinically translatable data. The consistent level of MBC/MIC ratio of 2.0 of all three agents depicts that these agents are really bactericidal and not merely bacteriostatic inhibitors. This property is especially relevant to caries management operations, where it is the total removal of *S. mutans* in the carious lesion and not just growth inhibitory effects that will produce the greatest likelihood of caries arrest and the avoidance of lesion reactivation [26]. The bactericidal capacity of the three agents favors their possible application as therapeutic agents in the treatment of active caries in primary teeth. The time-kill kinetics data is useful in providing the information about the speed of antimicrobial action which has clinical practice implication. High initial kill count of nano silver at 3-logs of viable counts in 4 hours indicates that comparatively short exposure durations can deliver significant antibacterial action. This has clinical applicability in the topical application situation where the agent is likely to be diluted and cleared by saliva upon application [27]. The reduced rate of killing with nano-chitosan, although efficient, implies that the preparations containing this substance could be improved by methods to increase the time of contact with the tooth surface, either by inclusion in a sustained-release system or adhesive varnish preparations. The possibility of using synergistic effect between these agents to achieve synergistic antibacterial effect is an exciting field of research in the future. The complementary action mechanisms between nano silver and nano-chitosan kind of actions, i.e., nano silver penetrates the cell through ion release and generation of reactive oxygen species, whereas the nano-chitosan formulations are able to disrupt the cell membrane due to electrostatic interactions, indicates that combination formulations might potentially be capable of attaining a greater efficacy at lower individual concentrations and therefore, the concern of potential cytotoxicity is reduced whilst still showing antimicrobial activity [28]. There are some shortcomings of this research. Although offering standardized and reproducible conditions, the *in vitro* design fails to recapitulate the multispecies biofilm environment that *S. mutans* resides within in the oral cavity that is a multispecies community embedded within an extracellular polysaccharide framework which can hamper antimicrobial penetration [29]. Planktonic susceptibility testing can be misleading (the clinical efficacy) because biofilm associated bacteria tend to be 100-1000 times more resistant to antimicrobial agents than their planktonic counterparts. The experiment tested the antibacterial effect against one cariogenic species, but the cariogenic process entails a polymicrobial community. There was no cytotoxicity evaluation of the human oral cells and the biocompatibility of these agents at efficient levels should be first determined before clinical translation. The test system could not have salivary proteins, acquired pellicle and other constituents of the oral fluid, which can influence the availability and activity of the antimicrobial agents in clinical conditions [30]. Future research needs to compare these agents to polymicrobial biofilm models, determine cytotoxicity, formulation and ultimately determine efficacy in clinical trials in pediatrics.

## Conclusion:

We show that nano silver, nano-chitosan and silver diamine fluoride exhibit significant antibacterial activity against Streptococcus mutans, supporting their role as anticariogenic agents in primary teeth. Nano silver showed the highest antibacterial efficacy, while silver diamine fluoride and nano-chitosan also demonstrated clinically relevant effects with additional advantages. Further in vivo and clinical studies are required to validate these findings and support their application in pediatric caries management.

## Figures and Tables

**Table 1:** Zones of inhibition (mm) Against *S. mutans* clinical isolate by agent and concentration (Mean ± SD, n = 9)

**Concentration**	**Nano Silver (AgNPs)**	**Silver Diamine Fluoride (SDF)**	**Nano-Chitosan (NCS)**	**p-value (One-way ANOVA)**
125 μg/mL (Low)	14.36 ± 1.28	12.48 ± 1.16	9.82 ± 1.34	<0.001
250 μg/mL (Medium)	18.62 ± 1.44	16.14 ± 1.28	13.26 ± 1.42	<0.001
500 μg/mL (High)	22.84 ± 1.62	19.76 ± 1.38	16.42 ± 1.54	<0.001
p-value (within agent, ANOVA)	<0.001	<0.001	<0.001	
Negative control (water)	0 ± 0	0 ± 0	0 ± 0	-

**Table 2:** Minimum inhibitory concentration (MIC) and minimum bactericidal concentration (MBC) values (μg/mL) against *S. mutans*

**Agent**	**Clinical Isolate MIC**	**Clinical Isolate MBC**	**MBC/MIC Ratio**	**ATCC 25175 MIC**	**ATCC 25175 MBC**
Nano Silver (AgNPs)	31.25	62.5	2	15.625	31.25
Silver Diamine Fluoride (SDF)	62.5	125	2	31.25	62.5
Nano-Chitosan (NCS)	125	250	2	62.5	125

**Table 3:** Time-kill kinetics at 2x MIC concentration: viable count (log₁₀ CFU/mL) over time (Mean ± SD, n = 6)

**Time Point**	**Control (No Agent)**	**Nano Silver (62.5 μg/mL)**	**SDF (125 μg/mL)**	**Nano-Chitosan (250 μg/mL)**
0 hours	5.72 ± 0.08	5.70 ± 0.10	5.71 ± 0.09	5.69 ± 0.11
1 hour	5.98 ± 0.12	4.86 ± 0.18	5.12 ± 0.16	5.34 ± 0.14
2 hours	6.34 ± 0.14	3.92 ± 0.22	4.38 ± 0.24	4.86 ± 0.20
4 hours	7.12 ± 0.16	2.68 ± 0.28	3.42 ± 0.26	4.14 ± 0.24
6 hours	7.86 ± 0.18	1.48 ± 0.34	2.36 ± 0.32	3.28 ± 0.28
12 hours	8.54 ± 0.14	0 ± 0	0.82 ± 0.46	1.86 ± 0.36
24 hours	8.92 ± 0.12	0 ± 0	0 ± 0	0.42 ± 0.38
